# Caffeine intake inverts the effect of adenosine on myocardial perfusion during stress as measured by T1 mapping

**DOI:** 10.1007/s10554-016-0949-2

**Published:** 2016-07-29

**Authors:** Dirkjan Kuijpers, Niek H. Prakken, Rozemarijn Vliegenthart, Paul R. M. van Dijkman, Pim van der Harst, Matthijs Oudkerk

**Affiliations:** 1 Center for Medical Imaging, University Medical Center Groningen, University of Groningen, Hanzeplein 1 EB 45, Groningen, The Netherlands; 2 Department of Cardiovascular Imaging, MCH-Bronovo, The Hague, The Netherlands; 3 Department of Cardiology, University Medical Center Groningen, University of Groningen, Groningen, The Netherlands

**Keywords:** T1-mapping, Cardiovascular MRI, Caffeine, Biomarkers

## Abstract

Caffeine intake before adenosine stress myocardial perfusion imaging may cause false negative findings. We hypothesized that the antagonistic effect of caffeine can be measured by T1 relaxation times in rest and adenosine stress cardiac magnetic resonance imaging (CMR), as T1 mapping techniques are sensitive to changes in myocardial blood volume. We prospectively analyzed 105 consecutive patients with adenosine stress perfusion CMR on a 1.5-T MRI system. Rest and stress T1 mapping was performed using Modified Look-Locker Inversion recovery. T1 reactivity was defined as difference in T1_rest_ and T1_stress_ (∆T1). Fifteen patients drank coffee within 4 h of CMR (<4H caffeine group), and 10 patients had coffee the day before (>8H caffeine group). Comparison was made to patients without self-reported coffee intake: 50 with normal CMR (control group), 18 with myocardial ischemia, and 12 with myocardial infarction. The national review board approved the study; all patients gave written informed consent. The <4H caffeine group showed inverted ∆T1 of −7.8 % (T1_rest_ 975 ± 42 ms, T1_stress_ 898 ± 51 ms, p < 0.0005). The >8H caffeine group showed reduced T1 reactivity (1.8 %; T1_rest_ 979 ms, T1_stress_ 997 ms) compared to the controls (4.3 %; T1_rest_ 977 ± 40 ms, T1_stress_ 1018 ± 40 ms), p < 0.0005. Ischemic and infarcted myocardium showed minimal T1 reactivity (0.2 and 0.3 %, respectively). Caffeine intake inverts the adenosine effect during stress perfusion CMR as measured by T1 mapping. T1 reactivity can assess the adequacy of adenosine-induced stress in perfusion CMR.

## Introduction

Myocardial stress perfusion cardio magnetic resonance (CMR) imaging is state of the art in the diagnosis of myocardial ischemia [1]. Although the sensitivity of adenosine stress perfusion CMR is reported to be over 90 % [2], results from the CE-MARC study suggested that over one-third of false negative studies may be related to insufficient pharmacologic stress mainly due to drug interactions [3]. The influence of caffeine on myocardial blood volume (MBV) is not exactly known. The primary mechanism of action of caffeine in humans occurs through the blockade of adenosine receptors [4] masking the adenosine induced vasodilatation and potentially diminish the sensitivity to detect perfusion defects. Secondly, caffeine increases sympathetic nerve activity, inducing capillary de-recruitment, which decreases myocardial perfusion reserve [5–8]. Myocardial capillaries are the primary determinant of myocardial blood flow (MBF), and closely reflect the level of microvascular sympathetic autoregulation [7, 8]. Heart rate and blood pressure correlate poorly with adenosine-induced hemodynamic changes, and cannot be used to assess the adequacy of stress testing [9].

Native (non-contrast) T1 mapping is a CMR technique used to quantify myocardial water content, closely related to MBV [10]. Native T1 can be used to discriminate between hypertrophic cardiomyopathy and hypertensive heart disease and it can be diagnostic in conditions associated with myocardial fibrosis, protein deposition and iron overload [11–14]. The increase of MBV during adenosine stress CMR can be measured by subtracting rest T1 values from T1 stress (∆T1). Recently, T1 mapping at rest and during adenosine stress has shown to be able to differentiate between normal, infarcted, ischemic and remote-dysfunctional myocardium [15]. We hypothesized that the antagonistic effect of caffeine to the adenosine effects during stress can be measured with T1 relaxation times at rest and stress using these new T1 mapping techniques.

## Materials and methods

### Patient selection

All subjects gave written informed consent to participate in the study and the national ethical committee granted all study procedures. Between August and December 2015 we prospectively enrolled 172 consecutive patients referred for stress perfusion CMR because of suspicion of myocardial ischemia in our institution. All patients avoided potential adenosine agonists for at least 24 h before CMR, and all anti-anginal medication was stopped 4 days before the examination. Dypiridamole had to be stopped and, if not possible, was considered a contraindication. At arrival in the CMR facility patients were specifically interviewed on the use of coffee. Patients were excluded from analysis based on general CMR contraindications [2], mapping motion artefacts [25] and technical failures [8]. To avoid T1 mapping measurement errors and interference with other diseases, all patients with underlying cardiomyopathy [18], or any combination of myocardial infarction, myocardial ischemia, and coffee [14] were excluded from analysis as well. Of the 105 remaining patients, 15 patients had caffeine accidentally containing coffee less than 4 h before the CMR study (<4H caffeine group), and 10 patients consumed coffee more than 8 h prior to CMR, generally taken the day (evening) before the study (>8H caffeine group). These groups were compared to 50 patients without myocardial abnormality at CMR (control group), patients with myocardial ischemia [18] and myocardial infarction [12] at CMR. Three patients in the <4H caffeine group were scanned twice (with and without coffee, with an interval of 2–6 weeks). One of these 2 patients (75 kg) received a much higher dose of adenosine (210 mmol/kg instead of 170 mmol/kg).

### Cardiac MR imaging protocol

A 1.5T system (Magnetom Avanto; Siemens Healthcare, Erlangen, Germany) was used in all patients. After the standard cine images, an investigational Modified Look-Locker Inversion Recovery (MOLLI) based T1–mapping sequence (WIP780B) was performed at rest and in stress. A 5(3)3 sampling scheme of the heart was performed, including 8 images in 11 heartbeats. For the MOLLI acquisition an initial Inversion time of 110 ms was used with an 80 ms increment. A pixel-wise T1 map of the myocardium was acquired in short-axis view with inline motion correction. The images were generated using a single-shot steady-state free-precession readout. Typical parameters were: field of view, 300 × 256 mm^2^; slice thickness 8 mm; acquisition matrix, 192 × 128; in plane spatial resolution, 1.4 × 1.4 mm^2^; bandwidth, 1085 Hz/pixel; flip angle, 35° and parallel imaging acceleration factor, 2. T1 maps were acquired at rest and during peak dose adenosine stress (140 µg/kg/min) in 3 short-axis slices (basal, mid-myocardial and apical). Stress-only perfusion imaging was performed as previously described, according to conventional methods [16]. In brief, a nonselective saturation recovery perfusion sequence was started during the first pass of 0.1 mmol/kg gadopentetate dimeglumine injected at a flow rate of 5 ml/s, after 3 min of adenosine infusion. Equal position of the 3 short-axis slices was used in T1 mapping, stress perfusion and late gadolinium enhancement (LGE). An additional dose of contrast material (0.05 mmol/kg) was given for LGE imaging.

### Image analysis

**Fig. 1:**
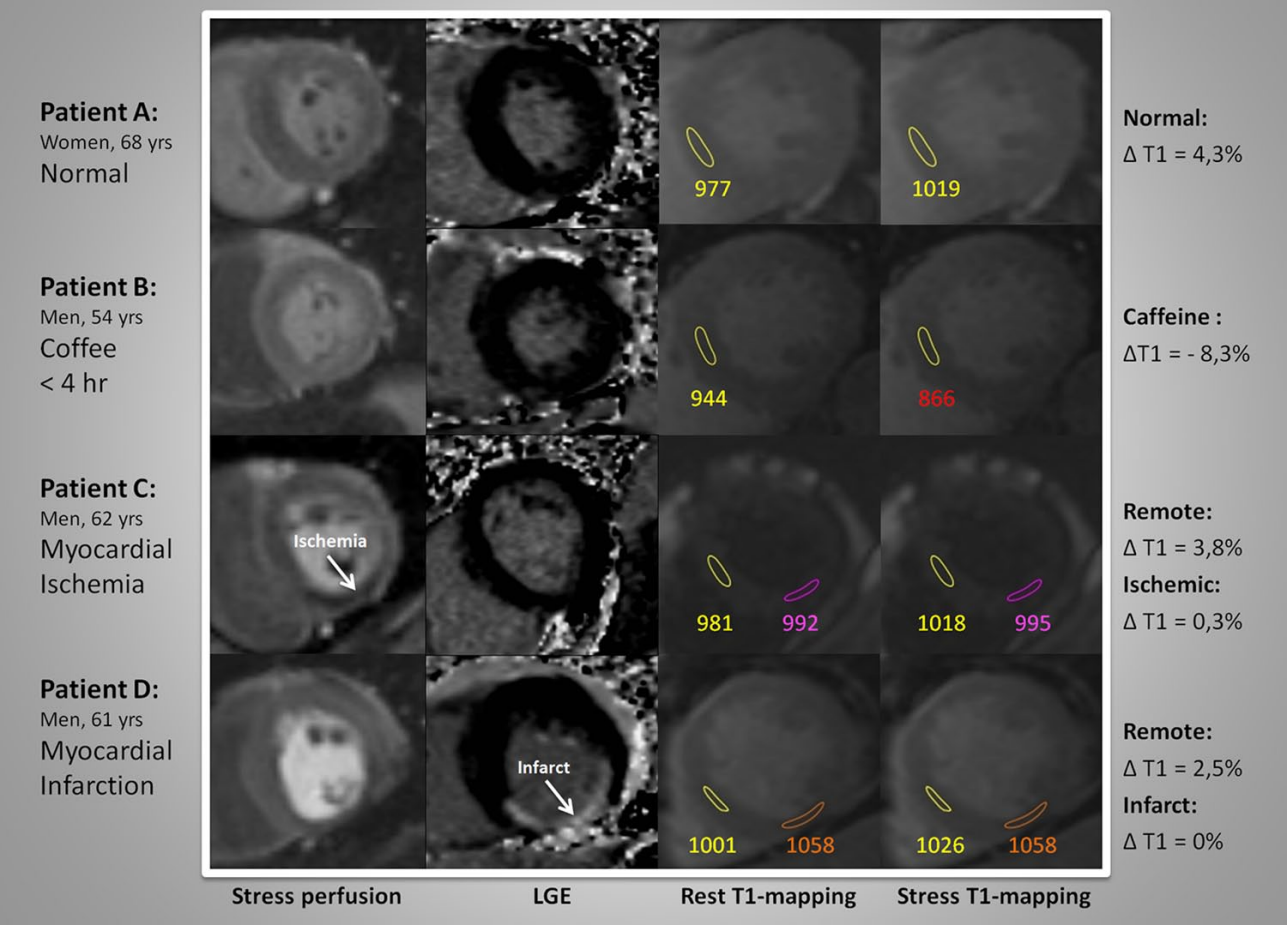
Rest-stress T1 mapping analysis with adenosine perfusion CMR

The T1 maps, generated on the imaging console, were analyzed on commercially available software (MASS analytical software, Medis, Leiden, The Netherlands). Short-axis T1 maps were manually contoured using conservative septal sampling [17], and specific sampling in ischemic or infarcted regions of interest (ROI) based on perfusion images and LGE images. See Fig. 1. To avoid partial volume effects of the blood pool, all samples were located in the core of the ROI. Remote myocardial ROI were placed carefully outside of the ischemic or infarcted region. The measurements of the T1 relaxation times were performed by two runs of quantitative measurements with MASS mapping software. The second run was blinded to the patient data. Perfusion and LGE series were visually analyzed as previously described [16]. Hemodynamically significant coronary artery disease was confirmed by invasive coronary angiography in all 18 patients with myocardial ischemia.

### Statistical analysis

All statistical analysis was performed using SPSS Statistics 23 (IBM Corporation, USA). Continuous values were presented as mean values (e.g. T1_rest_ and T1_stress_), and, if appropriate, ±standard deviation (SD). Categorical data were presented as percentages. Difference in T1_rest_ and T1_stress_ was expressed as percentage (∆T1 (%) = (T1_stress_− T1_rest_)/T1_rest_ × 100). Differences between two groups were tested by independent samples *t* test, and differences in T1 values between ischemic/infarcted myocardium and normal myocardium by paired *t* test. Differences between three or more groups (controls, <4H caffeine group, >8H caffeine group, ischemic (and remote) myocardium group, infarct (and remote) myocardium group) were assessed using one-way analysis of variance (ANOVA) including proper post-hoc testing based on the homogeneity of variances per measured variable. Values of P < 0.05 were considered statistically significant. SPSS Statistics version 22 was used.

## Results

Baseline characteristics and results of the 105 included patients are presented in Tables 1 and 2. As expected, heart rate and rate-pressure product increased during the infusion of adenosine (Table 1). Compared with patients in the control group, patients with coffee intake showed no significant difference in rate-pressure product increase (P > 0.05). The lowest increase in rate-pressure–product was seen in patients with infarction (1.5 %) and ischemia (4.4 %) (P > 0.05 versus controls).

**Table 1:** Characteristics of study objects

	Controls	<4H caffeine	>8H caffeine	Patients with ischemia	Patients with infarct	P value
	N = 50	N = 15	N = 10	N = 18	N = 12	
Male (%)	44	53	40	61	92	0.008
Age (years)	67 ± 11	67 ± 11	61 ± 15	71 ± 11	69 ± 10	0.325
Body weight (kg)	75 ± 14	79 ± 14	91 ± 23	83 ± 13	87 ± 19	0.013
History of PCI (%)	2	13	0	0	17	
History of CABG (%)	0	0	0	0	17	
Myocardial infarct age (months)	0	0	0	0	21 ± 16	
Resting heart rate, beats/min	76 ± 15	70 ± 7	77 ± 20	72 ± 10	77 ± 18	0.732
Stress heart rate, beats/min	87 ± 14	81 ± 10	88 ± 19	81 ± 19	87 ± 17	0.601
Increase in heart rate (%)	14	16	15	12	14	
Rest systolic blood pressure, mm Hg	146 ± 28	144 ± 20	143 ± 28	150 ± 25	161 ± 20	0.287
Rest diastolic blood pressure, mm Hg	85 ± 19	83 ± 9	78 ± 7	79 ± 9	87 ± 12	0.351
Stress systolic blood pressure, mm Hg	141 ± 23	139 ± 15	137 ± 23	138 ± 40	144 ± 20	0.989
Stress diastolic blood pressure, mm Hg	80 ± 10	79 ± 7	74 ± 7	74 ± 13	82 ± 10	0.069
Rest rate pressure product, mm Hg. beats/min	11,213	10,165	11,296	10,851	12,522	0.450
Stress rate pressure product, mm Hg. beats/min	12,320	11,357	12,277	11,334	12,715	0.821
Increase (%)	9.9	11.7	8.7	4.4	1.5	

Values are mean ± SD or percentages. Statistical difference between any of the groups tested by ANOVA testing.

*PCI* percutaneous coronary intervention, *CABG* coronary artery bypass grafting

**Table 2:** T1 mapping values

T1 mapping values	Controls	Coffee intake <4H	Coffee intake >8H	Patients with ischemia		Patients with infarct		P value
				Ischemic myocardium	Remote myocardium	Infarct myocardium	Remote myocardium	
	N = 50	N = 15	N = 10	N = 18	N = 18	N = 12	N = 12	
T1 mapping value at rest, ms	977 ± 40	975 ± 42	979 ± 37	979 ± 64	960 ± 44	1039 ± 49	988 ± 40	0.001
T1 mapping value at stress, ms	1018 ± 40	898 ± 51	997 ± 65	981 ± 61	985 ± 45	1042 ± 51	1018 ± 42	<0.0005
∆ T1 (% stress response compared to rest)	4.3 ± 2.8 %	−7.8 ± 5.0	1.8 ± 4.0	0.2 ± 0.7	2.6 ± 3.4	0.3 ± 0.4	3.1 ± 2.6	<0.0005

Values are mean ± SD or percentages ± SD. Statistical difference between any of the groups tested by ANOVA testing

**Fig. 2:**
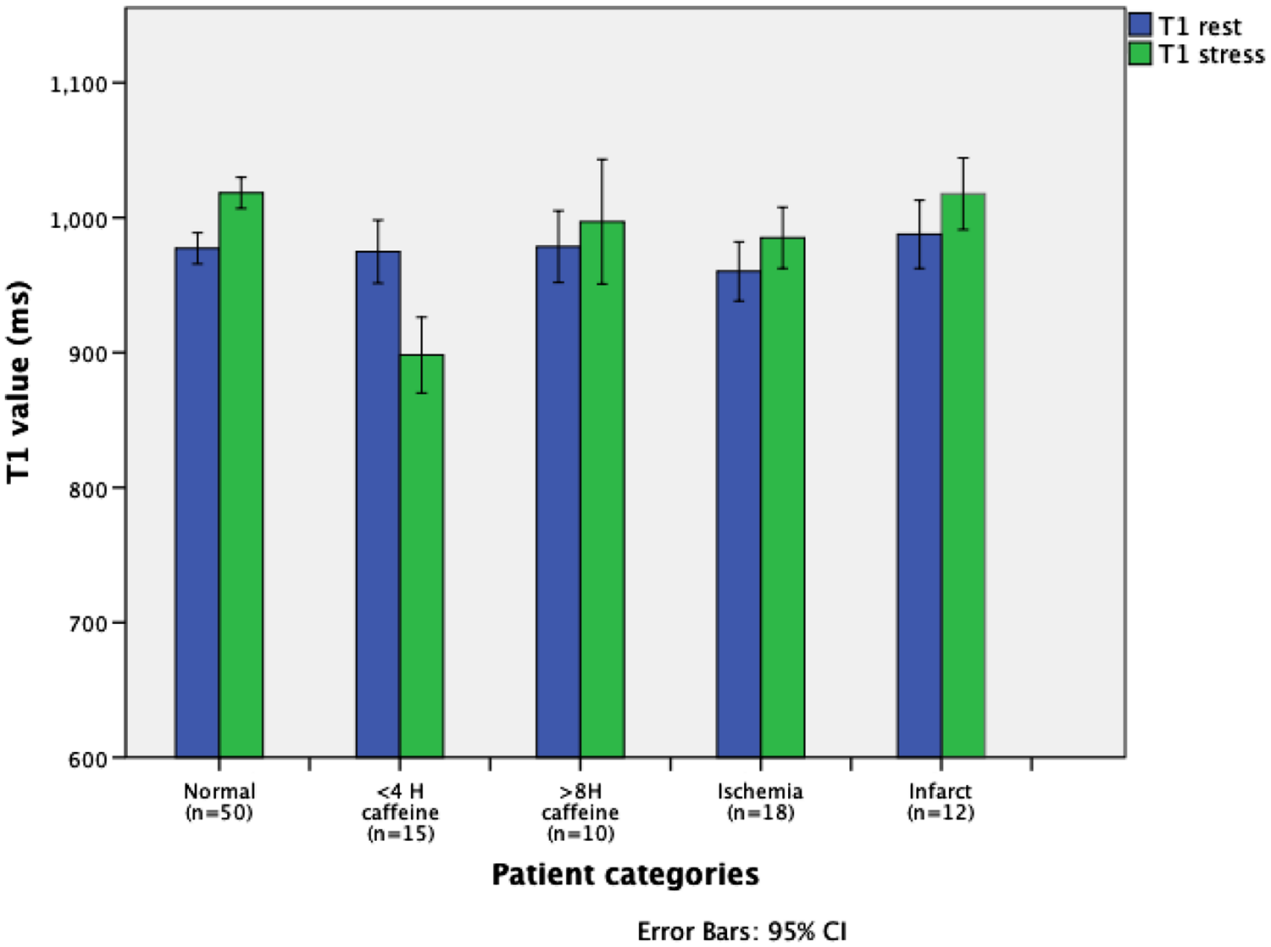
Myocardial T1 values at rest and during adenosine stress CMR

**Fig. 3:**
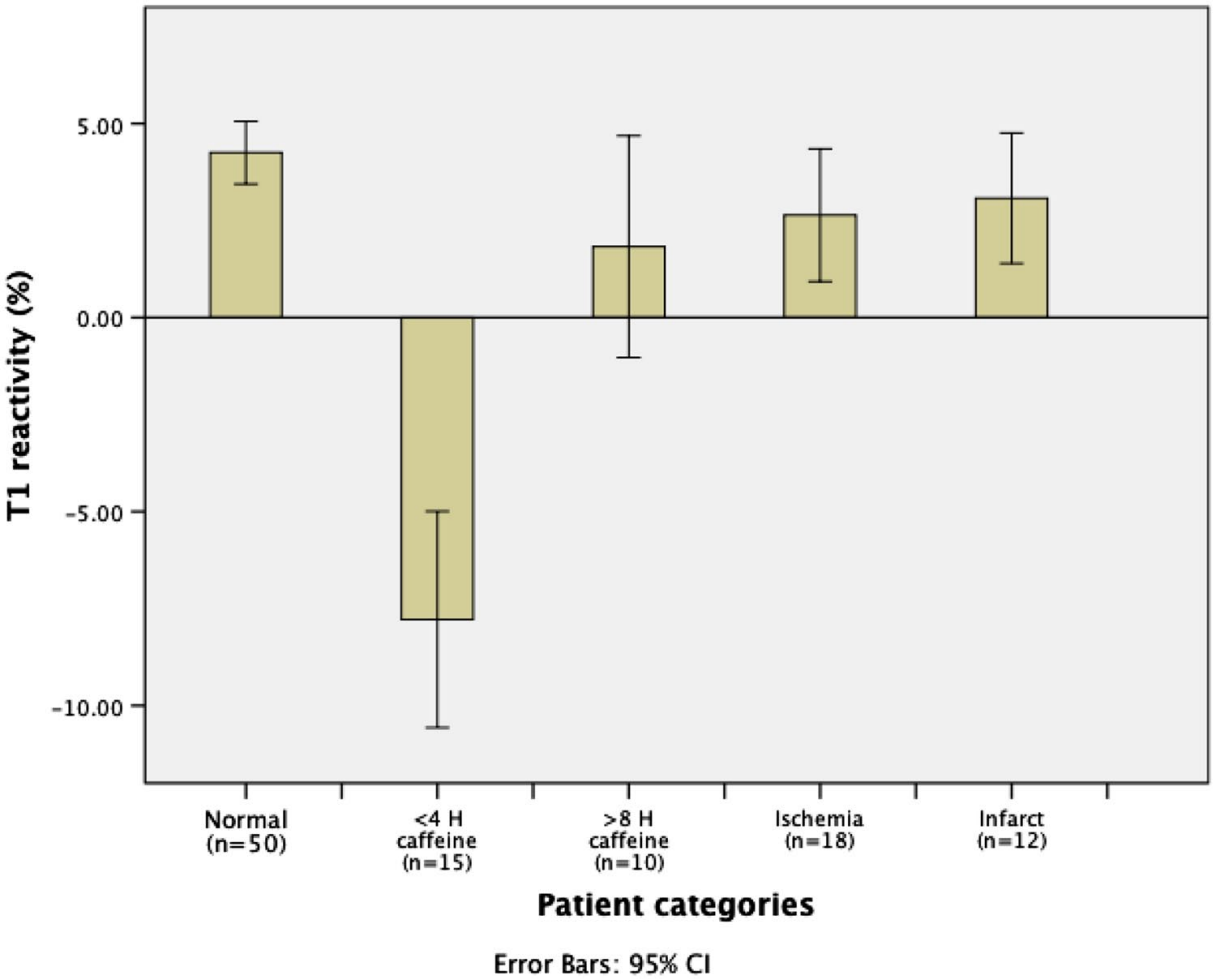
Comparison of T1 reactivity (∆T1) between the different patient groups

**Fig. 4:**
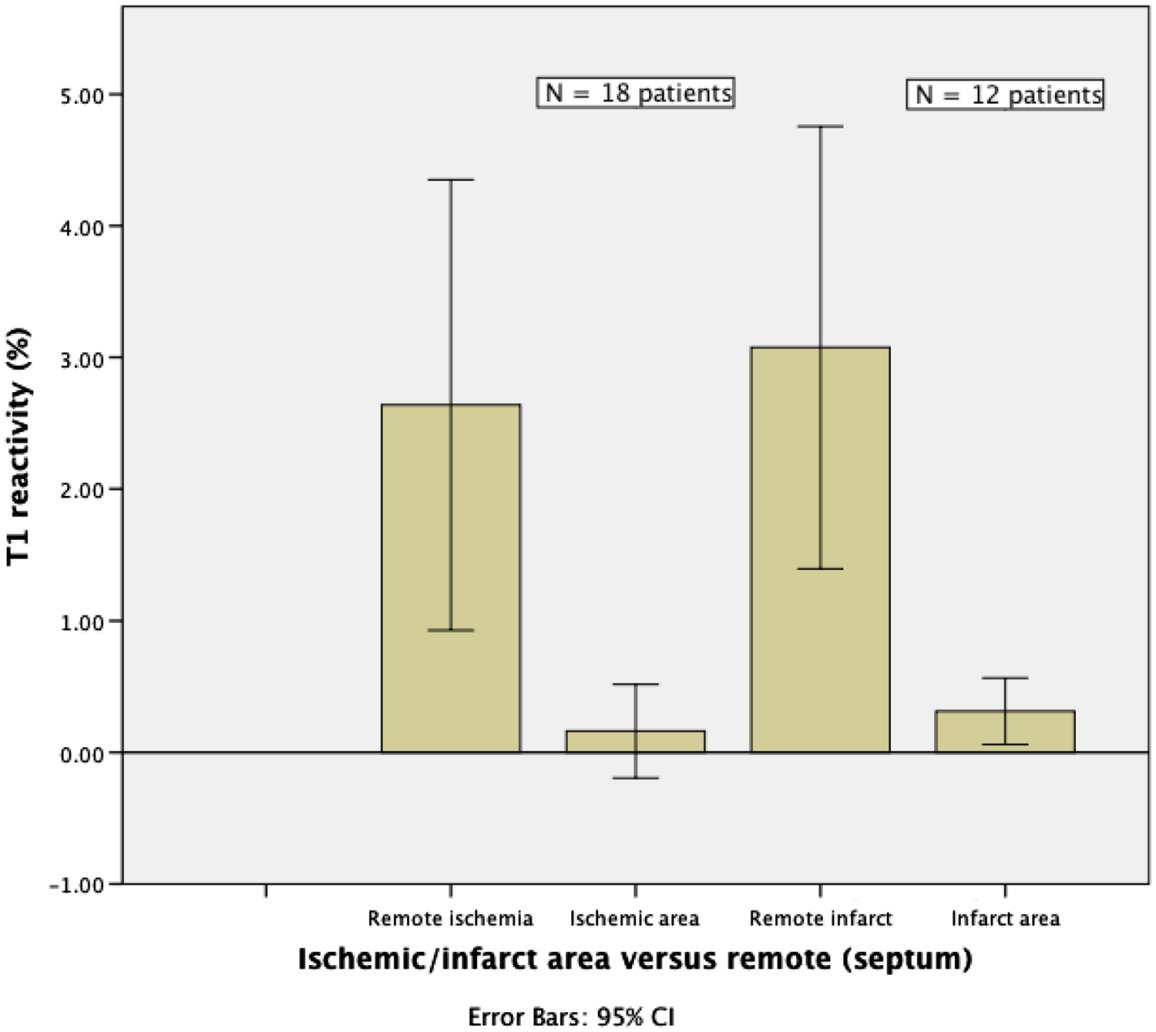
Comparison of T1 reactivity (∆T1) between ischemic, infarcted and remote myocardial tissue

**Fig. 5:**
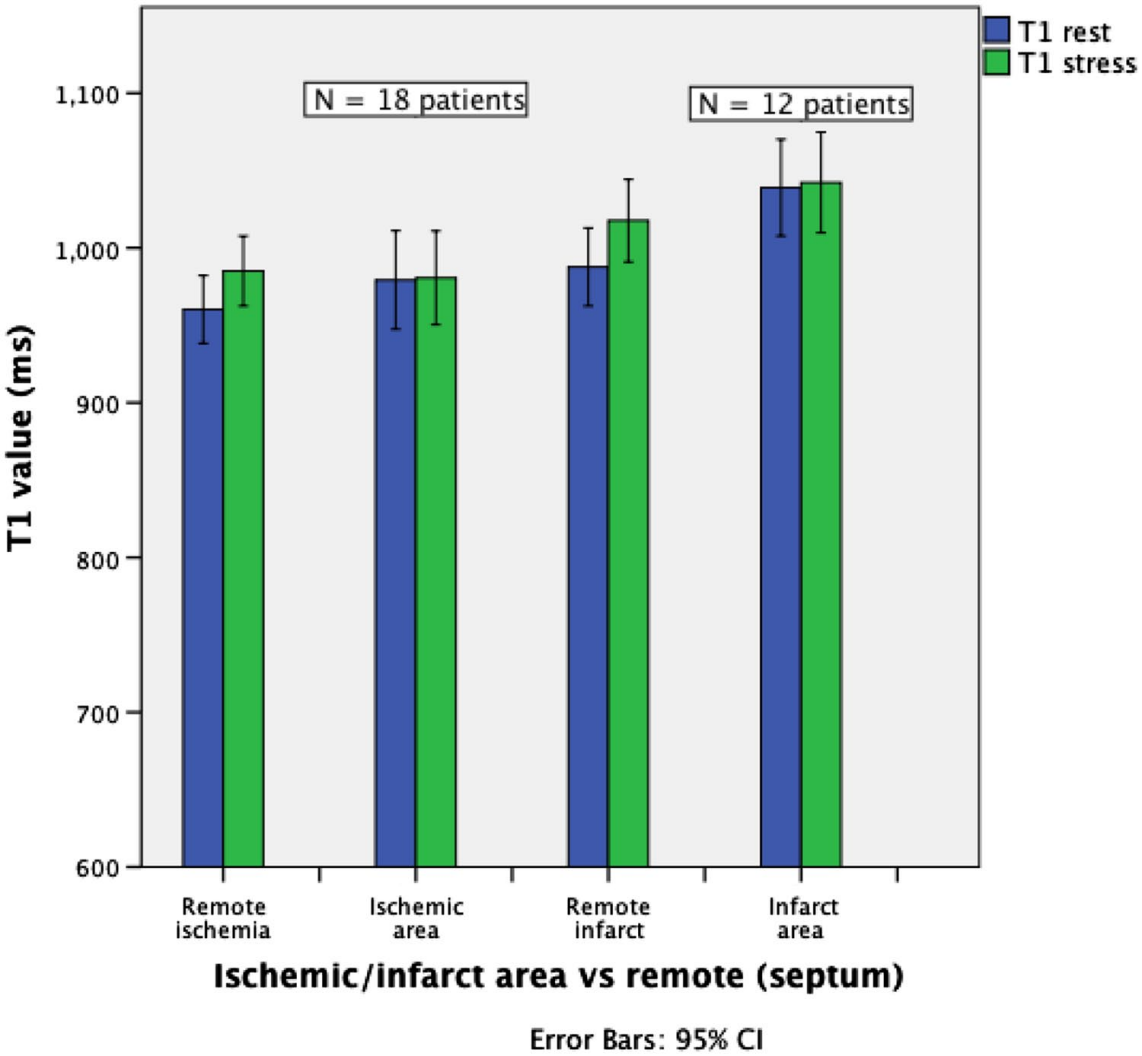
Comparison of T1 values in ischemic and infarct area versus remote myocardial tissue (septum)

The T1_rest_ value for myocardium in controls (977 ± 40 ms) was similar to the <4H and >8H caffeine group (975 ± 42 and 979 ± 37 ms, respectively), and to the ischemia group (979 ± 64 ms). The native T1_rest_ in the infarct group was significantly higher (1039 ± 49 ms) than in controls (P = 0.001). The ∆T1 was significantly lower in the <4H caffeine group as compared to all other groups (−7.8 %; P < 0.0005), including the >8H caffeine group (1.8 %) and the control group (4.3 %) (Figs. 1, 2, 3). A blunted ∆T1 was found in the remote myocardium in patients with ischemia (2.6 %) and infarction (3.1 %) (Figs. 4, 5). Ischemic (∆T1 0.2 %) and infarcted (∆T1 0.3 %) myocardium showed almost no T1 reactivity, the response being significantly lower as compared to controls (P < 0.0005, and P = 0.002, respectively).

The 3 patients in the <4H caffeine group, who had a repeated CMR stress study without coffee showed a positive ∆T1 of 4.2 %, which is similar to the control group. One of these 3 patients received the first study (with coffee) a much higher dose of adenosine (266 ml/h instead of 210 ml/h), which resulted in a ∆T1 of −16.5 %. In the repeated study the ∆T1 normalized to 2.5 %.

## Discussion

This study demonstrates that the increase of MBF of adenosine during myocardial stress perfusion is not only blocked, but inverted by coffee intake as measured by T1 mapping. We studied the short-term impact of caffeine consumption on T1-mapping and found that the ∆T1 values in the <4H caffeine group were significantly lower than all other ∆T1 values for all other groups in this study. Moreover there were no overlapping ∆T1 values between the <4H caffeine group and all other groups. This imaging biomarker is able to identify patients during the adenosine stress CMR study who are stressed inadequately due to caffeine, which may lead to false-negative perfusion test outcomes. ∆T1, or T1 reactivity, can be used as a benchmark for the adequacy of stress induction by adenosine at stress perfusion CMR studies and should be reported in the results.

We found no significant increase in T1_rest_ for caffeine groups versus the control group, which is comparable to studies that analyzed the effect of caffeine on MBF and MFR [5, 18]. The patients in the >8H caffeine group showed blunted T1 reactivity, but still significantly lower than the ∆T1 values found in the control group. The sympathetic nerve system plays an important role in the regulation of MBF [19]. Caffeine is a central nervous system stimulant of the methylxantine class and increases sympathetic activity and blood pressure [20, 21]. Furthermore, former studies reported that caffeine decreases exercise induced myocardial flow reserve (MFR), which is comparable to our findings [5].

In this study -in the absence of an infarction—an almost zero local ∆T1 was indicative for myocardial ischemia. Basic research has shown that the regulation of a constant capillary hydrostatic pressure precedes myocardial oxygen transfer [22]. Myocardial capillaries lack smooth muscle and hence do not dilate with adenosine. When normal myocardium is maximally dilated with adenosine, the capillaries are the bottleneck to hyperemic flow [23]. Even in the presence of a severe coronary stenosis the perfusion deficit is mainly due to capillary regulation and not to the stenosis itself [23]. Absence of capillary recruitment measured by ∆T1 mapping can therefore be indicative for myocardial ischemia, as shown in this study. In this regard it might be more suitable to use ∆T1 mapping as a reference standard in CMR perfusion studies concerning myocardial ischemia and not to use fractional flow reserve as provided by coronary angiography.

The T1_rest_ values of the infarct group as reported in this study (1039 ms) were lower than reported recently (1442 ms) [15]. These lower T1 values are unlikely due to manual drawing failures of the ROI because both studies used center of core samples to avoid partial volume effect of the blood pool. Nonetheless, the differences in T1 relaxation time are probably explained by the younger mean infarct age of the patients in our study (21 ± 16 months), which was substantially lower than those reported earlier (71 ± 70 months) [15].

The three patients who were imaged twice showed a tremendous recovery of ∆T1 after avoiding coffee for the stress test, which are in line with the main findings in this study. The results in this study supports the guidelines [1] that recommend avoiding caffeine for at least 24 h before the stress test. One of these patients received also a much higher dose of adenosine on the first study (with coffee). This patient showed a remarkable negative ∆T1 (−16.5 %) indicating severe capillary derecruitment and low MBF. Former reports indicate a MFR decrease of 22 % at rest and exercise in patients who consumed coffee within 1 hour before the stress study [5]. So, most likely, the major ∆T1 decrease of 16.5 % is comparable to a very low MBF, which could have serious clinical impact. In this regard this issue can be linked to the report of induced stress cardiomyopathy due to consumption of an energy drink containing high dose of caffeine [24]. This reverse stress cardiomyopathy (Takotsubo) is mainly seen in a younger age group and will continue to be an interesting topic in ischemic heart disease.

The hemodynamic data in this study showed no significant difference in rate-pressure products between the normal patients and the patients who consumed coffee. It is known that peripheral hemodynamic parameters during adenosine are poor indicators of changes of the MBF [9], which was expressed by the ∆T1 findings in this study.

### Strengths and limitations

A major strength of evaluating T1 reactivity by CMR, is the quantitative measure of response to adenosine. This allows more detailed assessment of the effect of adenosine than visual analysis of perfusion defects as used f.e. in SPECT. In fact, based on nuclear MPI-based studies there is debate about whether or not caffeine even exerts an effect on adenosine response [25, 26]. In contrast, even with the rather categorical information about coffee intake in our study, we were already able to find a very strong inverse effect on adenosine by timing of caffeine intake. Misclassification of caffeine intake would have resulted in bias towards the zero effect, which indicates the effect we describe may even be stronger with more detailed evaluation of caffeine level per patient.

The investigational MOLLI-based T1 mapping technique used in this study revealed a substantial number of motion artefacts, which could be overcome by using shorter independent heart rate T1 sequences. New applications of the used software may also correct these motions artefacts in the future.

In conclusion, rest-stress T1 mapping CMR can identify and quantify the antagonistic effect of caffeine on myocardial blood volume in patients stressed with adenosine. ∆T1 can be used as a benchmark for the validity of the stress induction by adenosine at cardiac MR perfusion studies. The results of this study are applicable on all types of adenosine myocardial perfusion imaging.
